# Supplementary material for: Complexome profiling on the Chlamydomonas lpa2 mutant reveals insights into PSII biogenesis and new PSII associated proteins
Source: J Exp Bot. 2021 Aug 26;73(1):245–62. doi: 10.1093/jxb/erab390 (PMC8730698; doi:10.1093/jxb/erab390)
Supplement: erab390_suppl_Supplementary_Dataset_S1 [file erab390_suppl_supplementary_dataset_s1.zip › Supplemental Dataset 1 - Excel List and all profiles/plots/MDN4_Cre12.g483950.html]

Trivial name: MDN4  
  
Euclidean distance: 766407.46  
Mean Intensity (WT): 94632.51  
Mean Intensity (Mut): 72234.97  
Distance: 8.10  
  
MapMan: TCA / organic transformation.TCA.malate DH;gluconeogenesis / glyoxylate cycle.malate dehydrogenase  
  
p value of intensity sums Welch test: 0.5379
